# Strategies for Digital Clinical Teaching During the COVID Pandemic: A Scoping Review

**DOI:** 10.1007/s40670-023-01894-w

**Published:** 2023-11-15

**Authors:** Miranda Voss, Anne Geniets, Niall Winters

**Affiliations:** 1 Department of Education, University of Oxford, Oxford, UK; 2 Harris Manchester College, Mansfield Road, Oxford, OX1 3TD UK

**Keywords:** Clinical teaching, Digital learning, Medical students

## Abstract

Widespread “lockdowns” during the COVID pandemic in 2020–2021 restricted medical students’ access to patients. We used a scoping review with exploratory thematic synthesis to examine how reports of digital clinical teaching during the first year of the COVID pandemic could inform digital clinical teaching in the post-pandemic world. We looked at strategies used and outcomes reported, lessons learned about how best to use digital methods for clinical teaching, and learning theories used. The eighty-three articles included in the final review fell into four groups. These were telehealth interventions; virtual case-based teaching; multi-modal virtual rotations; and a small group of “other” strategies. Telehealth reports indicated that COVID has probably accelerated the adoption of telehealth, and these skills will be required in future curricula. Engagement with virtual case-based teaching was problematic. Virtual rotations were particularly valued in specialties that relied on visual interpretation such as radiology and dermatology. For general clinical specialties, digital clinical teaching was not a satisfactory substitute for real clinical exposure because it lacked the complexity of usual clinical practice. Sixty-seven articles reported students’ reactions only, and 16 articles reported a change in knowledge or skills. Demands on instructors were considerable. Few studies were theorized and none tested theory, which limited their transferability. While telehealth teaching may be a valuable addition to some curricula, digital clinical teaching is unlikely substantially to replace exposure to real patients outside of specialties that rely on visual interpretation. High demands on instructors suggest little potential for new, scalable digital clinical offerings after COVID.

## Introduction and Background

In January 2020, the World Health Organization declared the novel coronavirus disease COVID-19 a Public Health Emergency of International Concern [1]. In March 2020, national lockdowns were announced in several countries and medical schools began restricting face-to-face teaching in order to reduce the risk of virus transmission. Clinical teaching provides an important case study of a move to online education because face-to-face contact between medical students and real patients has traditionally played such an important role in the training of a doctor.

Not surprisingly, a large volume of publications describing the responses of medical curricula to this challenge has been published. Gordon et al. [2] published a rapid systematic review in August 2020 that examined the literature published between the start of the pandemic and May 24, 2020. Forty-nine articles were included in their final analysis, selected from 7476 titles identified. Nearly half of these described developments in the undergraduate curriculum with 53% of articles describing curricula “pivoting to online education.” The papers reviewed discussed technical difficulties with online delivery of classes, behavioral conventions such as muting microphones, and how students could participate in online environments. As Gordon et al. point out, most of the papers they reviewed focused on praxis rather than evaluation. This was entirely appropriate early in the pandemic when changes to long-established teaching routines were rapidly required, but data about the educational consequences of the pivot to online clinical could be expected in publications written in the following year, and may offer important lessons for digital clinical teaching in a post-pandemic world. In this article, we present a structured review, based on scoping methodology, that examines the empirical literature on digital clinical teaching for medical students between June 2020 and May 2021, subsequent to Gordon’s systematic review. We asked the following review questions:

1. What strategies to provide online clinical teaching to undergraduate medical students were reported between June 2020 and May 2021 and what outcomes are reported?
2. What do these initiatives tell us about how best to provide clinical teaching using digital methods?
3. How did these authors theorize the use of digital media for teaching clinical reasoning?

## Methods

A scoping review [3] with an additional thematic synthesis [4] was used. Scoping reviews are part of the family of methods used for the systematic review of “messy” bodies of literature. They use standardized data extraction tables to map out evidence, and they are particularly valuable for describing the types of evidence found in a defined research field, examining key concepts, and identifying research gaps [3, 5]. However, document sets may also contain rich data about themes that were not defined *a priori* in the data extraction table, and an additional exploratory thematic synthesis [6] may be used to identify and organize themes relevant to the review question as they emerge from the data [4].

Most other types of structured review include a process of critical appraisal because the aim is to find the best available evidence to inform practice. In contrast, the purpose of a scoping review is to bring together and describe all the literature relevant to the research question, balancing comprehensiveness with feasibility. A formal assessment of methodological quality is therefore usually not made [3].

### Constructing the Search

Constructing a systematic literature search for social interventions, such as educational projects, is challenging because there is no widely used thesaurus of search terms equivalent to the Medical Subject Headings (MeSH) thesaurus used in medical research [7]. Systematic bibliographic searches are therefore likely either to return an overwhelming number of titles when specification is comprehensive, or to miss important literature when tightly specified [8]. Although more purposive search approaches have been developed to address this problem, such as “double sided snowballing,” which involves the tracking of concepts and empirical data from the citations to and references in key documents within the field [9], the literature of interest for the present enquiry is contained within a 12-month period. It was therefore decided to adapt the comprehensive bibliographic search terms specified by Gordon [2], omitting the search terms for postgraduate medical education, and student selection or assessment.

Although scoping reviews are often extended to include “gray” literature [3], the current search was limited to peer-reviewed publications in view of the volume of literature. The bibliographic databases MEDLINE, EMBASE, CINAHL, and PsychINFO were searched using the following search terms:

(coronavirus OR covid19 OR covid-19 OR SARS-Cov-2) AND (Medical education OR undergraduate medical OR medical student OR medical school OR clinical skills OR clinical teaching).

### Inclusion and Exclusion Criteria

The boundaries of a scoping review are defined by population, concept, and context [3]. The following limits were applied:

- *Population*: Medical students
- *Concept:* English language primary literature reporting
  - Digital methods used to teach clinical skills during the first year of the coronavirus pandemic
  - Some sort of outcome or evaluation
  - Clinical skills, including skills to elicit clinical findings and make sense of them. Also didactic instruction about clinical problems. Instruction in technical skills is excluded.
- *Context:* Formal medical school curricula. No geographical limiters applied
- *The following exclusion criteria were defined:
*
  - *Commentaries:* no evidence of primary or secondary research. Includes editorials, reflections, and narrative reviews. However, perspectives written by medical students were considered relevant and screened in. Also excluded: descriptive empiric work that measures attitudes to online learning and teaching in general.
  - *Clinical care/service delivery:* Including issues around infection control, public health and also COVID research, team training for COVID, public knowledge.
  - *GME/CME:* Graduate medical education and continuing medical education. Articles about COVID impact on medical student career decisions; virtual conferences; recruitment to specialist programs; faculty development.
  - *Praxis:* “How we did it,” recommendations for learning design and descriptions of use/delivery of materials without evidence of primary empirical data. Also, praxis of educational research.
  - *Non-clinical learning*: Medical student teaching that is not directly clinical, e.g., medical sciences, public health, evidence-based healthcare.

### Article Selection

**Fig. 1:**
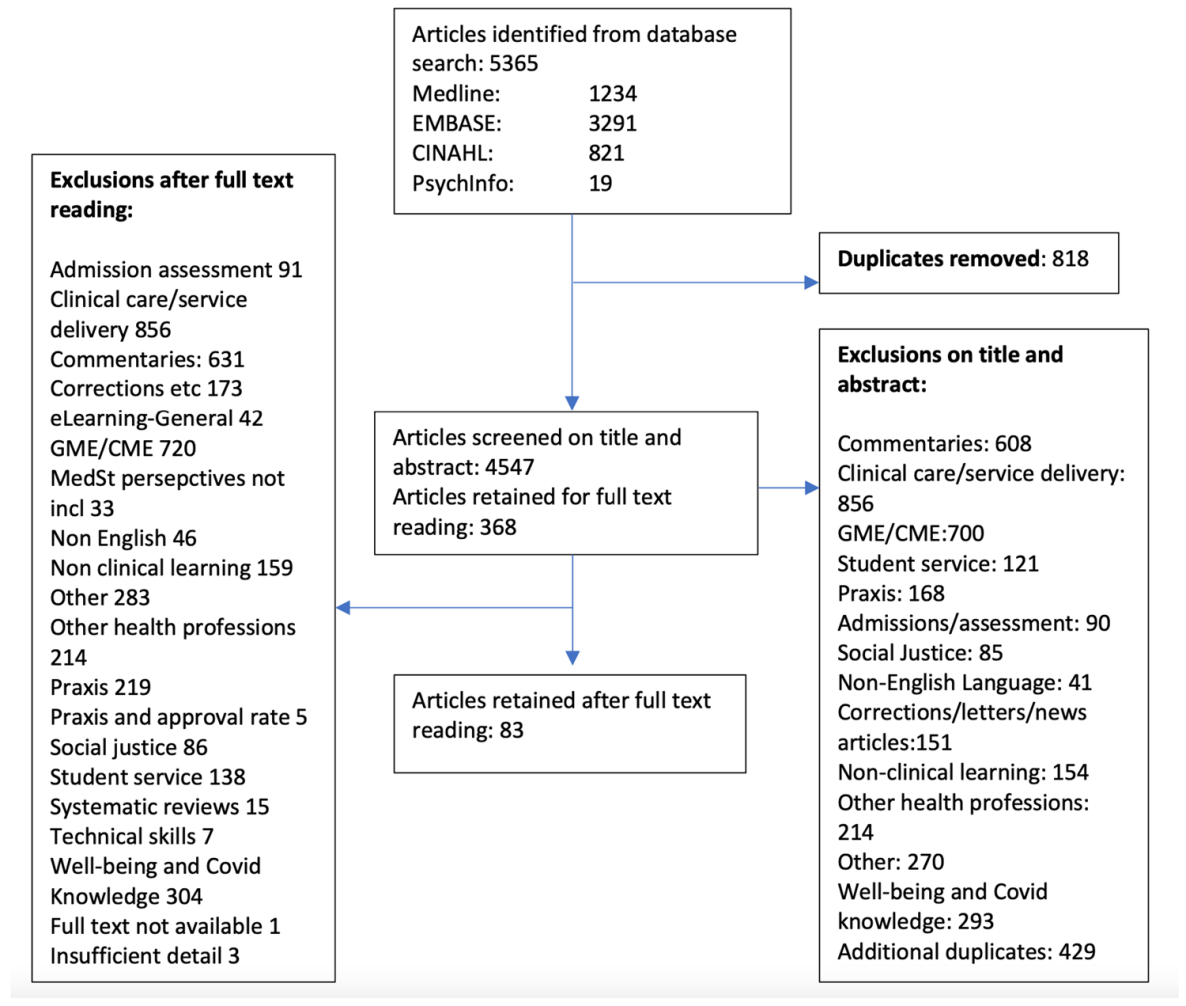
PRISMA chart showing selection of articles for this review

Citations were imported into EndNote for deduplication and screening on title and abstract by a single author. Full text for the selected citations was downloaded and the articles were read for inclusion. A PRISMA chart showing the process of article selection is shown in Fig. 1.

### Analysis

The selected full-text articles were imported into Atlas-ti Version 9.1 (ATLAS ti Scientific Software GmbH, Berlin). Reports of similar strategies were grouped together in an iterative fashion for ease of analysis. Separate data extraction tables were created for each strategy. Documents grouped under similar strategies were also read as whole and open-coded [10] for themes relevant to the review questions, with a particular emphasis on students’ reactions to the programs and learning preferences.

## Results

### Review Question 1: What Strategies to Provide Online Clinical Teaching to Medical Students Were Reported Between June 2020 and May 2021 and What Outcomes Are Reported?

Eighty-three documents reported 82 programs that provided substitutes for clinical teaching. The three dominant strategies emerged from the process of document sorting, these were as follows: telehealth programs (*n*=22); multi-modality virtual rotations (*n*=31), and virtual case-based teaching (*n*=16). Fourteen “other” strategies were identified; these predominantly involved didactics. The strategies used in these document groups are summarized in Tables 1, 2, 3, and 4.

**Table 1:** Programs that used telehealth for clinical teaching

**Article**	**Country**	**Types of patients**	**Telehealth context**
Abraham *et al.* 2020 [53]	USA	Real	Internal medicine ambulatory care
Adams and Ecker [14]	USA	Real	Various, not specified but ambulatory
Aron *et al.* 2020 [16]	USA	Real	Pandemic follow-up clinic
Ashrafzadeh *et al.* 2020 [54]	USA	Real	Dermatology outpatients
Bautista *et al.* 2020 [55]	USA	Real	Interprofessional telehealth outreach to at-risk patients. with pharmacy team
Brem *et al.* 2021 [56]	Switzerland	Standardized^a^	Course in telephone consultation for medical emergencies
Cain *et al.* 2020 [57]	USA	Real	Family medicine consultations
Cannon *et al.* 2021 [15]	UK	Student role play	Training for family medicine telehealth consultation
Carson *et al.* 2020 [58]	USA	Real	Telehealth consultations: students staffed telephone “hotline” for underserved communities
Darnton *et al.* 2021 [12]	UK	Real	Telehealth consultations family medicine
Harendza *et al.* 2020 [59]	Germany	Standardized	Telehealth training
Hartmann *et al.* 2021 [60]	Germany	Standardized	Telehealth training (focus on communication)
Hayes *et al.* 2020 [61]	USA	Real	Family medicine consultations
Ho *et al.* 2021 [62]	USA	Real	Intensive care. Students accessed video and EMR information from patient’s room
Knie *et al.* 2020 [42]	Germany	Role play and standardized patients	Telehealth training (focus on communication)
Lalchandani *et al.* 2021 [63]	USA	Real	Telephone outreach to geriatric patients
Martinez *et al.* 2020 [64]	USA	Standardized	Telehealth training
Ross *et al.* 2021 [65]	USA	Role play	Telehealth training (sexual history taking)
Safdieh *et al.* 2021 [13]	USA	Real	Telehealth rotations in a number of specialities
Sukumar *et al.* 2021 [36]	USA	Real	Internal medicine. students accessed EMR and rounds. Opportunity to discuss and present
Tsang *et al.* 2021 [38]	Hong Kong	Real	Students interviewed (?one) inpatient using telehealth
Weber *et al.* 2021 [17]	USA	Real	Outpatient telehealth consultations

*EMR* electronic medical record

^a^Standardized patients are actors or members of the public who have been trained to play the role of a patient with a specific problem

**Table 2:** Programs that used curated cases to provide digital clinical teaching

**Article**	**Field**	**Country**	**Strategy**
Alpert *et al.* 2020 [66]	Radiology	USA	Faculty supervise small groups for virtual reads using video-conferencing
Alrasheed *et al.* 2021 [67]	Interprofessional education	Saudi Arabia	Interprofessional students worked collaboratively on a case using video conferencing
Chadha *et al.* 2021 [68]	Ophthalmology	USA	Reasoning session on the red eye using video-conferencing
De Ponti *et al.* 2020 [69]	Medicine and surgery	Italy	Online digital simulated cases that allowed interaction (synchronous, moderated by tutor)
Fatani 2020 [45]	Pediatrics	Saudi Arabia	Case-based discussion using videoconferencing
Foo *et al.* 2021 [21]	Surgery	Hong Kong	Case-based discussion using video conferencing. PBL format
Furlan *et al.* 2021 [70]	Medicine (single case)	Italy	Virtual patient simulator using natural language processing (Focus more on technological proof of concept)
Grant *et al.* 2021 [71]	Psychiatry	USA	Synchronous large group didactics using video conferencing, smaller group breakouts for case discussions
Osborne *et al.* 2021 [47]	Emergency medicine	UK	Cases based on reality TV
Rahm *et al.* 2021 [18]	Not specified	Germany	Asynchronous virtual cases
Rosenthal *et al.* 2021 [19]	Emergency medicine	USA	Short didactics followed by case-based discussion using video conferencing
Rullmann *et al.* 2020 [72]	Cardiology	Germany	Case-based discussion using video-conferencing. Audio files for auscultation findings
Sanseau *et al.* 2021 [40]	Emergency medicine	USA	Simulation using non-interactive, pre-recorded video and discussion using video conferencing
Wells *et al.* 2020 [73]	N/A	USA	Simulation using standardized patient Practice of data collection and patient “hand over” using video conferencing
Yang *et al.* 2021 [74]	Pediatrics	USA	Simulation using standardized parent and scripted clinical findings
Zottmann *et al.* 2020 [20]	Not specified (cases taken from New England J Med)	Germany	Case-based discussion using video-conferencing

**Table 3:** Programs that used multiple methods to produce a digital clinical rotation

**Article**	**Specialty**	**Country**	**Patient contact**	**Strategy**
Adams *et al.* 2021 [23]	Radiology	USA	No	Online e-readings Links to existing materials Video-conferencing lectures and discussion
Afonso *et al.* 2020 [41]	Introductory respiratory medicine	USA	Standardized patients	Clinical examination taught by video conferencing Telemedicine encounter with standardized patient Discussion and reasoning using video conferencing
Alamer and Alharbi 2021 [75]	Radiology	Saudi Arabia	No	Series of online didactics and tutorials using video conferencing
Armon *et al.* 2021 [76]	Obstetrics and gynecology	Israel	No	Asynchronous didactics Synchronous case discussions and didactics using video conferencing
Belfi *et al.* 2021 [77]	Radiology	USA	No	Digital messaging system (WhatsApp) Asynchronous preparation using LMS Synchronous case discussions, didactics, and read-out sessions using video conferencing
Boulger and Onello 2020 [31]	Rural family medicine	USA	No	Case discussions, journal club, panel discussions all using video conferencing
Byrnes *et al.* 2021 [33]	ENT-head and neck surgery	USA	Real	Synchronous didactics and case discussions using video-conferencing Telemedicine encounters with real patients Live-stream video from operating room using head mounted camera and wireless audio communication
Coffey *et al.* 2020 [34]	A number of core specialities	USA	Telehealth included in 2 of 6 programs	Rotations in 6 different specialities with different components
DePietro *et al.* 2021 [78]	Interventional radiology	USA	No	Synchronous case discussions and didactics using video conferencing Asynchronous preparation and didactics using LMS
DeVaro *et al.* 2020 [79]	Ophthalmology	USA	Real (optional)	Asynchronous preparation and didactics Synchronous peer to peer teaching using video conferencing, synchronous faculty lead case discussions Telemedicine encounters with real patients (optional) Electronic chart reviews
Divatia and Friedland 2020 [80]	Internal medicine and pediatrics	USA	No	Video-conferencing didactics, case-based, and mentoring discussions Additional learning materials hosted on cloud drive
Djermester *et al.* 2021 [81]	Not specified	Germany	No	Online (“video and podcast”) tutorial followed by paper-based clinical reasoning exercises
Durfee *et al.* 2020 [24]	Radiology	USA	No	Asynchronous preparation and didactics Videoconferencing large group lectures Small group workshops and case-based discussions using video conferencing
Geha and Dhaliwal 2020 [46]	Internal medicine	USA	No	Asynchronous podcasts and assignments Synchronous case discussions using video conferencing
Gomez *et al.* 2020 [22]	Radiology	USA	No	Synchronous and pre-recorded didactics using video conferencing Case-based workshops using video conferencing Asynchronous materials, including quizzes, on LMS Virtual “office hours” Journal discussion board
Kahn *et al.* 2021 [82]	Radiation oncology	USA	Real	Didactics and case-based learning on LMS Synchronous case discussions using video-conferencing Telemedicine observation
Kasai *et al.* 2021 [83]	Internal medicine and respiratory medicine	Japan	Simulated	Simulated cases loaded onto LMS and discussed later using video-conferencing. Also physician role play for history taking
Monday *et al.* (2020) [25]	Mixed	USA	No	Virtual “bootcamp” for internship preparation. Synchronous, interactive didactics Asynchronous review of procedural videos Asynchronous virtual cases
Pettitt-Schieber *et al.* 2021 [84] Kuo *et al.* 2021 [85] (This abstract reports one of the 8 rotations described above)	Eight surgical specialities	USA	Limited real	Video conferencing for: Student and faculty delivered didactics and Grand Rounds Mentoring sessions Operative videos Case-based learning Virtual skills lab Telemedicine
Phillips *et al.* 2021 [28]	Dermatology	UK	Webinars included talks from patients	Team-based messaging platform used for: Synchronous case-based discussions and journal clubs Asynchronous didactics – webinars and quizzes (? Synchronous) sessions with patient educators
Sandhu *et al.* 2020 [27]	Radiation oncology	USA	Real	Synchronous lectures and chart rounds using video-conferencing Multidisciplinary “tumor boards” using video conferencing Telemedicine observation Journal club using video conferencing
Shin *et al.* 2020 [44]	General surgery	USA	Resident role play	Case discussions via video-conferencing Follow-up didactics and operative videos Practice presentations in virtual table rounds
Smith and Boscak 2021 [29]	Radiology (trauma and emergency)	USA	No	Asynchronous didactics using open access resources Case-based teaching using stored images (self-review with later video conference) Student presentations (video conferencing)
Steehler *et al.* 2021 [30]	ENT	USA	No	Asynchronous didactic material Synchronous didactics (lectures and case-based learning) using video conferencing Synchronous discussions Student presentations
Suematsu *et al.* 2021 [86]	Interprofessional education	Japan	Standardized patients and family members	Online asynchronous didactics Synchronous discussions using video conferencing
Topor *et al.* 2021 [37]	Physical medicine and rehabilitation	USA	No	Asynchronous didactics Synchronous case discussions, journal club, and presentations
Traba *et al.* 2021 [87]	Clinical skills	USA	Resident role play	Case-based discussions using video conferencing and virtual simulation
van der Keylen *et al.* 2020 [43]	Family medicine	Germany	No	Asynchronous virtual didactics and assignments Synchronous discussion using video conferencing Hybrid elective on decision making
Vielsmeier *et al.* 2020 [32]	ENT	Germany	No	Synchronous and asynchronous didactics Asynchronous interactive virtual cases with synchronous discussion via video conferencing
Williams *et al.* 2021 [26]	Urology	USA	No	Asynchronous didactics Synchronous discussions and case presentations using video conferencing Student evidence-based medicine presentation using video conferencing

*LMS* learning management system

**Table 4:** Strategies for digital clinical teaching not included in the three major categories

**Article**	**Specialty**	**Country**	**Patient contact**	**Strategy**
Anteby *et al.* 2021 [48]	General surgery	Israel	No	Development of a series of didactic podcasts on clinical surgical topics
Austin *et al.* 2021 [88]	Emergency medicine	USA	Simulation using manikins	Small number of medical students called into a hybrid face-to-face/remote simulation with small group debriefing
Bala *et al.* 2021 [89]	Not specified	UK	Real	Mixed reality ward round of two patients using holography and digital communication platform
Castro *et al.* 2021 [90]	Variety but 53% internal medicine	USA	Some projects included telemedicine	Students participated in a number of instructor-designed clinical projects in order to meet clinical competencies
Chandrasinghe *et al.* 2020 [91]	Surgery	Sri Lanka	No	Didactics and case discussions using video conferencing, asynchronous discussion forums using Facebook
Conway-Jones *et al.* 2020 [92]	Critical care	UK	No	COVID-related didactics using video conferencing
Do Phuoc *et al.* 2020 [93]	Orthopedics	Vietnam	No	Discussion, demonstration, and practice of knee examination
Dow *et al.* 2020 [35]	Family medicine	UK	Real	Students shown video recordings of authentic consultations in primary care
Fischbeck *et al.* 2020 [94]	N/A	Germany	Student role play	Largely asynchronous course on the medical conversation with some video conferencing
Kopp *et al.* 2021 [95]	Infectious diseases and nephrology	USA	Real	Supervised students responded to consultation requests within the electronic medical record
Rose *et al.* 2021 [96]	Ophthalmology	India	No	Students shown series of videos of eye examination
Schleicher *et al.* 2021 [39]	N/A	Germany	Student demonstrated examination on another person at home	Didactic instruction on examination of musculoskeletal and neurological systems
Situ-LaCasse *et al.* 2021 [97]	Point of care ultrasound examination	USA	Standardized patients	Online didactics for point of care ultrasound scan followed by in-person evaluation of image acquisition skills
Suppan *et al.* 2021 [98]	Management of stroke	Switzerland	No	Users randomized to either traditional video or interactive, asynchronous module to learn a stroke scale

The reported outcomes were assessed using Kirkpatrick’s 4-level model, which is widely used in reviews of medical education research [11]. However, this model offered little discrimination, with 68 of 84 articles classified as Kirkpatrick level 1 outcomes (students’ reaction). Nine articles were classified as level 2 (change in knowledge or skill) and 7 articles reported outcomes at both levels 1 and 2. One additional article reported numbers of podcasts downloaded. Most of the level 1 outcomes reported were the results of student feedback, which usually showed appreciation for the online module, while most level 2 outcomes reported the change in test scores or self reported confidence after the module. Five articles used a comparison group who had been exposed to a prior face-to-face offering. These were as follows: a report of a radiology rotation, in which questions about the student experience generally favored the digital offering [66]; a radiology rotation, in which students had similar test results to the pre-pandemic cohort [24]; a virtual rural health rotation in which course evaluations were less positive than the pre-pandemic cohort [31]; a digital problem-based learning activity in which students performed less well in the digital version [21]; and a simulated patient consultation in which no difference was found in the student experience [59]. Just 5 of the reports used formal qualitative methods to assess the student experience, although several included data provided from open-ended feedback questions.

### Review Question 2: What Does This Body of Literature Tell Us About How to Use Online Methods for Clinical Teaching?

Although most of the reported results consisted only of course evaluation scores and an increase in students’ knowledge following teaching, there was a great deal of rich information contained in the descriptions of course design, implementation, and the authors’ reflections. Several themes emerged across the document set, and these are most appropriately examined within the strategy groups that emerged from the analysis.

#### Telehealth Strategies

Twenty-two documents used remote consultation, either by video link or telephone, to teach clinical skills. These reports are summarized in Table 1. Of the 15 articles from the USA, 13 exposed students to remote consultation with real patients. All but two of these involved either primary care or an ambulatory setting and, in several of them, students provided a service for patients: taking “hotline” telephone calls; accessing at risk patients; or helping patients to use technology before consultation with their supervising faculty. Of 6 articles from Europe/the UK, only one reported remote contact with real patients; the other programs had a focus on teaching telecommunication skills and used either role play or standardized patients (actors). The following major themes emerged from the document analysis in this group.

##### COVID Has Accelerated the Move to Telehealth

There was a strong sense that ambulatory consultations are moving towards telehealth and that curricular changes during COVID had accelerated this move and embedded telehealth in the curriculum:

Student Z4013 ‘I think it was good experience to do video consultations, especially as it might be going towards that in the future.’ [12]

Previously, the majority of the training at our institution focused on the mechanics and logistics of tele-health encounters and use of standardized patients for simulated telehealth encounters. Real-time development and implementation of a telehealth program for medical students with more applied application of telehealth skills was borne out of necessity due to COVID-19 [13]

in July 2020, when our students returned to their in-person clinical rotations, students continued to engage in telehealth across all specialties, who were utilizing telehealth in very diverse ways [14]

##### Different Skills Are Required During Remote Consultation

A number of articles reported the challenge of collecting clinical data during a telehealth consultation. Many noted the inability to perform a virtual physical examination and the difficulty in picking up non-verbal cues from patients, although some stated that this may have caused them to pay more attention to verbal communication:

It was noted by students that by not being able to directly see the patient in front of them, they had to be ‘more in tune’ with the clinical need so as not to overlook it. [15]

Students also valued the way it forced them to have to take better histories when they could not examine the patient or even (in some cases) visualise them:

Student 9349 ‘... having to adjust to asking questions that you would have found out the answers for in an exam, but asking the patient the questions instead, which is good I think practical medical experience ... ’ [12]

##### Demands on Supervisors

The move to telehealth clinical rotations was clearly demanding on faculty and resources at a time when their own clinical responsibilities had changed. The telehealth clinical rotations with real patient contact required preparatory courses for students, the development of tools such as scripts, supervision of the students, and the selection of suitable patients for student consultation.

Faculty reported high levels of stress in learning to care for patients and provide high- quality care through this unfamiliar modality. They described a lack of tools and time to consider how a student might be integrated into the virtual environment. [14]

Under the supervision of an attending physician, we conducted follow-up calls with patients referred by emergency department staff, triage tent providers, and triage phone line operators evaluating possible COVID-19 cases. Between March 17 and April 10, 2020, we completed 2,176 calls with 1,009 unique patients...The attending [consultant] joined the patient calls to discuss symptoms, answer questions, and confirm 1 of 3 dispositions: referral to in-person evaluation, ongoing phone follow-up, or discharge. Teaching occurred in one-on-one encounters with the attending and in-group reflection sessions. [16]

The importance of faculty training and technical support to deal with this was highlighted by several programs [14, 41, 75].

##### Telehealth as an Access Barrier

Although telehealth is seen as a technology that will reduce barriers to care by removing transportation difficulties and reducing the time associated with attending a conventional outpatient appointment, it may reduce access to care in at-risk populations:

Social determinants of health were frequently recognized as barriers to successful tele-health delivery for underserved patients. Technological illiteracy was often cited in association with elderly patients. [17]

There were also socially determined barriers to the use of telehealth in the student body:

Our results describe the considerable lengths to which students went to make suitable practical arrangements for home-based consulting. However, the arrangements described are commonly dependent upon socially determined elements (for example, private living space, control over environment, fast internet connection etc.) [12]

## Virtual Case-Based Teaching

Sixteen articles reported the use of curated cases for digital clinical teaching, which were based on either text and image based, or used simulation. These are summarized in Table 2. Fifteen of the 16 projects offered synchronous, moderated teaching. A variety of technologies was used to present clinical details to the students. Although these were often simple clinical descriptors, two used interactive online simulations and one used cases taken from reality television. The following concerns emerged from the qualitative document analysis.

### Authenticity

It was important to students and course designers that online cases should be realistic:

The practical context of the cases helped to make learning more realistic (S13, C1: ‘The case places you directly into clinical routine. You have really the feeling to oversee the case. The proximity to reality is present i[n] this concept’. Embedding of clinical pictures and features made it realistic; S31, C1: ‘Through pictures everything was more realistic’) [18]

[Of peer led sessions]

peers could teach most of the ‘didactic’ material with a focus on what is emphasized at their training level; and residents could then provide more depth when needed, caveats, and ‘real-life’ examples to make the material come alive, and be available to answer questions [19]

## Engagement and Interactivity

Engagement with learning was an issue:

Participation in facultative evaluation dropped from 164 (82.8%) in the beginning of the semester (Case 1 –chest pain) to 98 (49.5%) at the end of semester (Case 10 – musculoskeletal pain). Length of free-text answers declined from Case 1 to 10 [18]

While the subjective learning gain was significant, it was low compared to results of a previous study of CCD [Clinical Case Discussion] in a face-to-face setting with on-site attendance [3] ... phenomena such as ‘Zoom fatigue’ [12] occur in association with digital teaching, which may have had a negative impact on the students’ self-assessment. [20]

Several strategies were used to encourage active student participation: these usually involved interaction with faculty and with each other:

cognitive activations were implemented to prevent lurking (i.e., passive participation) of individual participants [6]: Specifically, during step 1 of dCCD [digital Clinical Case Discussions], students were required to take notes on the case presentation. After a short pause for reflection, all students sent a draft assessment to the moderator individually, which was discussed in step 2. The students also worked out the differential diagnosis dyadically or triadically in so-called ‘breakout rooms’ (a function in Zoom where the conference can be split into separate sessions) and posted it in the chat, which in turn served as a basis for step 4. [20]

Each tutorial lasted for two hours and was considered sufficient for students to go through two scenarios, discuss the relevant history and physical examination findings, decide on the suitable investigations, come up with working diagnosis and suggest the appropriate management [21]

### Multi-modal Rotations

In this group of 31 documents, authors used a variety of strategies and technologies to produce a digital version of an existing clinical rotation. The strategies used are summarized in Table 3

Several of the themes in this document set reproduced the concerns raised in reports on telemedicine and online case-based learning. There was a strong emphasis on interactivity and engagement, particularly with didactic sessions:

In addition to Zoom, several resident-led sessions utilized the Nearpod platform, which allows the presenter to embed assessments and interactive polls into lecture slides. [22]

Students could unmute their microphone to ask or answer questions, however the chat box proved to be a fast and efficient method to incorporate audience interactivity [23]

However, concerns about digital fatigue were prominent, particularly regarding online lectures:

Students were asked to provide suggestions for improvements to the virtual radiology course. The most frequent responses included limiting the didactic lectures to 1 hour and adding audience response questions to every didactic lecture. [24]

[Student feedback]

‘If you kept these lectures online, I would suggest shortening/separating them if possible. As a student, it is hard to pay full attention online with multiple hour-long lectures.’ [25]

#### Virtual Learning Offered an Opportunity for Less-Represented Specialties

Several reports saw the shift to digital learning as an opportunity to establish their specialty more strongly in the conventional curriculum:

Finally, this format could correct the pervasive lack of Urology (and surgical subspecialties) within pre- clinical curriculums. [26]

These data from our students are valuable to enhance the incorporation of radiation oncology into the general medical school curriculum. [27]

This was particularly the case for fields that rely on visual interpretation, such as radiology and dermatology:

Radiology, in particular, is well suited as a specialty for blended learning. Our images are easily accessed via the internet and generally do not require dedicated interaction with a patient [23]

Dermatology, already marginalized in traditional curricula relative to patient burden, has been disrupted, potentially due to an historical reliance on brief face-to-face (F2F) encounters to visualize dermatoses… Although online communities cannot – and should not – replace patient contact, supplementary exposures can improve confidence in skin examination [28]

#### Shared or Re-used Materials

Several programs used publicly available materials for didactics:

Students were directed to review a variety of publicly available online radiology education resources covering a broad range of topics including appropriate utilization and ordering of imaging studies, radiological anatomy, and interpretation of common imaging studies (reinforced during the interactive sessions), and to review selected publicly available unknown cases. [29]

Or were planning to re-use their recorded course materials in the future:

Each day the lectures were archived for viewership among our students and any other student of the Emory medical community [30]

### Other Approaches

Fourteen articles reported the use of a diverse set of strategies that are summarized in Table 4.

### Themes Appearing Across Document Sets

There were several themes that appeared across the four document groups:

#### Student Reactions to Digital Learning Were Influenced by the Necessities of the Pandemic

There was evidence that students appreciated the use of digital substitutes when face to face teaching was not possible:

Students commented that their preference would be for direct community base experiences, but were appreciative of the efforts and adjustments required due to the pandemic. [31]

Students expressed gratitude for the opportunity to continue learning despite the world around them grinding to a halt. [22]

This means that reactions to and use of digital resources during periods of social distancing may not indicate likely learning behaviors when face-to-face learning resumed:

Even though the evaluations were positive in terms of content, the frequency of utilization and also the motivation for feedback seems disappointing. This seems to be associated above all with an increasing return to everyday life after the end of the lockdown. [32]

And there was a strong feeling that digital methods could not replace face to face teaching:

Another thought the virtual elective was an ‘excellent option’ given the situation but that it ‘cannot and should not replace a real rotation’ [33]

One reason was that online teaching lacked real world complexity:

Online teaching cases just do not capture the complexity and learning value of seeing real patients. [34]

They recognized that uncertainty exists in many consultations and that there may not be a right or wrong answer in every case. [35]

It is unlikely that any remote form of education will replace the multifaceted education provided by in-person clerkships. [36]

And widespread concern that physical examination skills were not being learned:

competencies in the physical exam, procedural skills, relating to patients, and outpatient clinic experience were the most negatively impacted by the virtual curriculum. [37]

From this experience, we learned that only the detection of physical signs is considered to have been hampered by web-side teaching. [38]

#### Instructor Student Ratios

High ratios of instructors to students were apparent:

2 tutors for 4 course participants were provided, so that on one hand the tutor could demonstrate the examination technique at one person and on the other hand each tutor could concentrate on only 2 students when correcting the learned technique. [39]

Each drill was run by cofacilitator dyads, with medical student groups ranging in size from four to 10 learners per remote session. [40]

.Allocating ‘mentors’ to students aimed to provide one-to-one interaction with a faculty member, ensuring concerns could be raised privately and faculty development supported. [28]

Online lectures usually required a second faculty member to moderate the chat room, and small group discussions were valued:

The strength most frequently noted by the students was the small group homeroom sessions. Students felt that the home- room format was interactive and fostered an engaging learning environment. [24]

[student response] ‘Small group setting is also great. I feel like I get a lot more out of these sessions compared to in-person ones that are larger.’ [41]

### Review Question 3: How Have These Authors Theorized the Use of Digital Media for Teaching Clinical Reasoning?

Eight of 83 reports drew on formal theory. The theories used were as follows: Social Learning Theory and Community of Practice [28]; Hargie’s Communication as Skilled performance [42]; Self-determination theory and Mayer’s Personalization Principle [43]; Constructivism, social and humanism learning theories and Situated Learning [44]; Community of Enquiry [45]; Social Learning theory [46]; Anchored Instruction [47]; and Cognitive Apprenticeship [36, 46].

Theory was exclusively used to justify or inform instructional design. No clear evidence was found in this document set of empirical data being used to test or develop theory about digital learning.

## Discussion

Many medical schools worldwide reacted rapidly and imaginatively to the COVID pandemic and provided a range of virtual substitutes for clinical teaching, almost certainly accelerating the move to digital platforms for some aspects of medical education. The literature included in this review provides a “snapshot” of the strategies that were used in this global health emergency, and offers a synthesis of the lessons that can be drawn from these, mostly short-term, interventions.

Skills for clinical data collection were notably provided by telehealth experiences, which were the only strategies that involved real patients. In other interventions, student or instructor role play, manikins, standardized patients, or text descriptions were used as patient substitutes in order to help students to build their own libraries of illness scripts. One of the striking features of the non-telehealth approaches is the demand by students for highly interactive digital tools, small group discussions, and real-time feedback. The few projects that provided resources for asynchronous use tended to report a decline in use over time, particularly following a return to face-to-face teaching [18, 32, 48]. The work that went into creating and delivering these learning opportunities, particularly those that relied on intensive virtual coaching is striking, particularly given that many of the faculty will have been concomitantly managing an increased clinical workload.

Telehealth programs, probably deserve to be considered as a special case. Before the pandemic, there were existing pressures to use technology for remote consultation with patients who have complex needs and mobility issues and the pandemic has pushed this agenda forward [49]. However, despite the fact that telehealth was the only modality consistently to offer students authentic patient contact, it was not without problems. Firstly, it was clear that a different set of communication skills is needed for teleconsultation and the inability to examine a patient can be problematic. The use of telehealth also represented an access barrier, not only for patients who are less digitally literate, but also for students wishing to access telehealth consultations from homes that may not have quiet space, home internet access, or adequate bandwidth.

One of the great appeals of digital teaching is its scalability, and the potential to develop shareable learning materials, which may be particularly useful for less well-resourced institutions [50]. Exploration of this set of documents suggests that, far from levelling up learning, technology may widen disparities in higher education. The teaching strategies that were well received required good bandwidth; quiet, private study areas; and enough faculty to moderate small group discussions and answer questions. Conversely, the types of resources that are scalable—asynchronous didactics and assignments—are less well received and less used. Students simply do not like them, and this raises the question: why not? Theory was used sparingly in these reports, and the use of formal theory was limited to prescriptions for learning design. Under-theorization is an important criticism of medical education research in general [51] and this necessarily limits our understanding about how students learn and why they use resources in particular ways.

“Zoom fatigue” is a term that emerged during the pandemic to describe the feeling of exhaustion that follows prolonged video-conferencing. Although there is limited empirical work on “Zoom fatigue,” Bailenson et al. [52] drew on psychological theory to argue that it results from the stress associated with close-up interaction with other people’s faces and of looking at one’s own face for hours on end, and the cognitive load associated with giving and interpreting non-verbal cues. However, Zoom fatigue does not explain the particular lack of engagement with asynchronous, self-learning digital resources. Another potential explanation of learning engagement found in this document set was emotion. Osborne et al. [47] reported that using cases from reality television provoked emotional experiences that the students described as a “hook for learning,” or which made the teaching more memorable. How digital clinical teaching can be designed in general to provoke positive emotional responses in students is an open question and an interesting avenue to explore.

A better understanding of the mechanisms that explain engagement with online learning, particularly asynchronous learning, is needed. Without better strategies to engage online learners in asynchronous, scalable offerings, disparities between institutions may deepen in an increasingly digital world.

## Data Availability

The primary data reviewed in this study is available from the publishers.
